# The effects of sucrose and arsenic on muscular insulin signaling pathways differ between the gastrocnemius and quadriceps muscles

**DOI:** 10.3389/fendo.2023.1165415

**Published:** 2023-05-09

**Authors:** Pablo Pánico, Myrian Velasco, Ana María Salazar, Patricia Ostrosky-Wegman, Marcia Hiriart

**Affiliations:** ^1^ Department of Cognitive Neurosciences, Instituto de Fisiología Celular, Universidad Nacional Autónoma de México, Mexico City, Mexico; ^2^ Department of Genomic Medicine and Environmental Toxicology, Instituto de Investigaciones Biomédicas, Universidad Nacional Autónoma de México, Mexico City, Mexico

**Keywords:** insulin resistance, skeletal muscle, GLUT4, arsenic, metabolic syndrome

## Abstract

**Introduction:**

Insulin resistance in muscle can originate from a sedentary lifestyle, hypercaloric diets, or exposure to endocrine-disrupting pollutants such as arsenic. In skeletal muscle, insulin stimulates glucose uptake by translocating GLUT4 to the sarcolemma. This study aimed to evaluate the alterations induced by sucrose and arsenic exposure in vivo on the pathways involved in insulinstimulated GLUT4 translocation in the quadriceps and gastrocnemius muscles.

**Methods:**

Male Wistar rats were treated with 20% sucrose (S), 50 ppm sodium arsenite (A), or both (A+S) in drinking water for 8 weeks. We conducted an intraperitoneal insulin tolerance (ITT) test on the seventh week of treatment. The quadriceps and gastrocnemius muscles were obtained after overnight fasting or 30 min after intraperitoneal insulin injection. We assessed changes in GLUT4 translocation to the sarcolemma by cell fractionation and abundance of the proteins involved in GLUT4 translocation by Western blot.

**Results:**

Male rats consuming S and A+S gained more weight than control and Atreated animals. Rats consuming S, A, and A+S developed insulin resistance assessed through ITT. Neither treatments nor insulin stimulation in the quadriceps produced changes in GLUT4 levels in the sarcolemma and Akt phosphorylation. Conversely, A and A+S decreased protein expression of Tether containing UBX domain for GLUT4 (TUG), and A alone increased calpain-10 expression. All treatments reduced this muscle’s protein levels of VAMP2. Conversely, S and A treatment increased basal GLUT4 levels in the sarcolemma of the gastrocnemius, while all treatments inhibited insulin-induced GLUT4 translocation. These effects correlated with lower basal levels of TUG and impaired insulin-stimulated TUG proteolysis. Moreover, animals treated with S had reduced calpain-10 protein levels in this muscle, while A and A+S inhibited insulin-induced Akt phosphorylation.

**Conclusion:**

Arsenic and sucrose induce systemic insulin resistance due to defects in GLUT4 translocation induced by insulin. These defects depend on which muscle is being analyzed, in the quadriceps there were defects in GLUT4 retention and docking while in the gastrocnemius the Akt pathway was impacted by arsenic and the proteolytic pathway was impaired by arsenic and sucrose.

## Graphical Abstract

Male Wistar rats consuming 20% sucrose and 50 mg/L sodium arsenite for 8 weeks developed systemic insulin resistance. In quadriceps muscles, the main alterations were low expression of TUG after arsenite treatment and low expression of VAMP2 induced by all treatments. In gastrocnemius, all treatments reduced insulin-stimulated GLUT4 translocation by different pathways. Arsenite impaired Akt phosphorylation and TUG proteolysis, while sucrose inhibited TUG proteolysis and capn10 expression. Created with BioRender.com

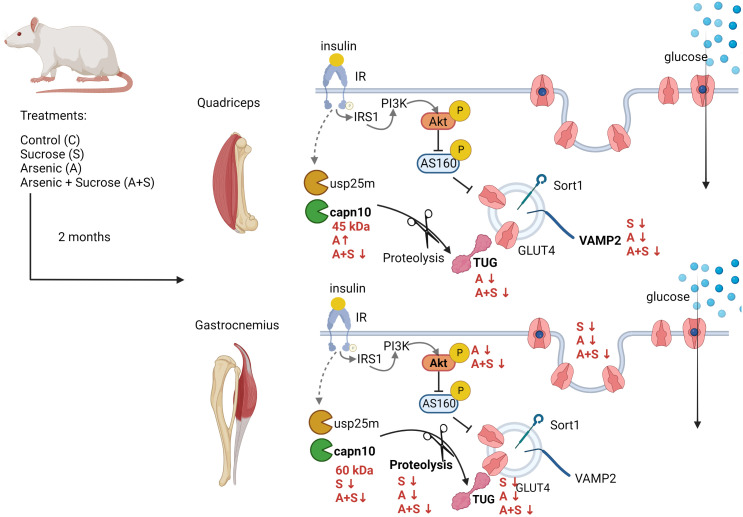

## Introduction

Metabolic syndrome (MS) is a condition characterized by at least three of the following signs: insulin resistance, central obesity, impaired fasting glucose, hypertension, and dyslipidemia. It increases the risk of developing type 2 diabetes (T2D), non-alcoholic fatty liver disease, cardiovascular diseases, and some forms of cancer (1). Classical risk factors for this condition include a sedentary lifestyle and consuming hypercaloric diets often including sweetened drinks (1). In addition, there is increasing evidence that exposure to endocrine-disrupting chemicals, such as arsenic, raises the risk of developing the signs of MS (2). However, the possible interaction between diet and arsenic exposure on the development of the signs of MS is poorly characterized.

Arsenic is an environmental pollutant distributed worldwide that can be produced by natural and anthropogenic sources (3). Exposure to arsenic through drinking water is associated with a higher risk of developing T2D and MS (4–8). Moreover, arsenic exposure induces systemic and muscle insulin resistance *in vivo* and *in vitro*, resulting in structural damage of muscle and loss of lean body mass (9–12). Specifically, arsenic inhibits insulin-stimulated glucose uptake (ISGU) by decreasing insulin-induced translocation of the glucose transporter-4 (GLUT4) to the plasma membrane in muscle fibers and adipocytes *in vitro* (12–15). Nevertheless, most of these studies focused on phosphorylated levels of Akt, whose alterations are known to have minimal impact on insulin-stimulated GLUT4 translocation in skeletal muscle (16, 17). At the same time, arsenic’s effects on other insulin signaling steps remain largely understudied.

Skeletal muscle is a central tissue coordinating the body’s energy balance; in the presence of insulin, it is responsible for up to 80% of whole-body glucose disposal (18). Moreover, muscle insulin resistance results in poor glycemic control and is a primary factor in determining the development of T2D (19). Mechanistically, activation of insulin receptor (IR) induces its transphosphorylation at several tyrosine residues, which in turn triggers different signaling pathways controlling ISGU, glycogen synthesis, lipid accretion, protein synthesis, and muscle growth (18). Regarding ISGU, IR activation promotes GLUT4 mobilization from the intracellular GLUT4 storage vesicles (GSV) to the sarcolemma and T-tubules (18) by activating several pathways. These include 1) the recruitment and activation of the phosphatidyl inositol 3 kinase (PI3K), inducing the synthesis of phosphatidyl inositol 3,4,5 triphosphate (PIP3), and activating protein kinase Akt (18). Active Akt phosphorylates and inhibits the proteins TBC1D1 and TBC1D4 (also known as AS160) in muscle, which are negative regulators of GLUT4 translocation (18). 2) The proteolytic pathway, involving the activation of the proteases usp25m and calpain-10 (capn10), which cleave the Tether containing UBX domain for GLUT4 (TUG) protein, releasing the GSVs from their perinuclear location (20, 21). The coordination between these pathways is necessary to effectively translocate GLUT4 to the sarcolemma and induce ISGU (22, 23). However, there is evidence that alterations of the IRS/PI3K/Akt/TBC1D1/4 axis are not a principal cause of muscle insulin resistance, and recent works focus on pathways independent of this axis (17, 21).

Skeletal muscle is a highly heterogeneous tissue of different proportions of oxidative and glycolytic myofibers, satellite cells, connective tissue, vascular cells, and neuron axons. The interactions between these components determine each muscle bed’s mechanical and metabolic properties, resulting in different responses to pathophysiological conditions, including insulin resistance (24–26). Notably, a specific muscle bed’s response during pathological conditions does not depend only on fiber composition, and muscles with similar fiber compositions differ in their response to environmental cues (25). Thus, comparing the defects in insulin signaling between multiple muscle groups is needed to determine how each muscle responds to environmental cues.

Previously, we reported that a model of MS in Wistar rats consuming 20% sucrose through drinking water for 2 months developed central obesity, insulin resistance, cardiac arrhythmias, hypertriglyceridemia, and alterations in pancreatic beta-cells (27–30). Thus, we aimed to evaluate the alterations induced by sucrose and arsenic consumption *in vivo* on the canonical and proteolytic pathways that control insulin-stimulated GLUT4 translocation in the quadriceps and gastrocnemius muscles.

## Materials and methods

### Animals and treatments

All animal protocols involved in this study were approved by the Animal Care Committee of the Instituto de Fisiología Celular, Universidad Nacional Autónoma de México (UNAM; CICUAL MHU189-22). Animal care was performed according to the International Guiding Principles for Biomedical Research Involving Animals, Council for International Organizations of Medical Sciences, 2010. The animals used for this work are part of a more extensive study aimed at identifying the mechanisms induced by arsenic and sucrose that contribute to the development of MS. Most of their tissues and organs were collected for several analyses.

A total of 104 young male Wistar rats (250-280 g, approximately 8 weeks of age, 26 rats for each condition) were obtained from the local animal facility of IFC, UNAM. The animals were housed in a cycle of 12 hours of light and 12 hours of darkness, at 20-23°C and 40% relative humidity. The rats were randomly assigned to each of the experimental conditions: control (C), maintained with tap water; sucrose (S), treated with 20% sucrose in drinking water; arsenic (A), treated with 50 ppm of sodium arsenite in drinking water; arsenic + sucrose (A + S), treated with 20% sucrose and 50 ppm of sodium arsenite in drinking water. Although this arsenite dose is considerably higher than the environmentally relevant concentrations for human populations, it is important to note that rats are resistant to the toxic effects of arsenic (3). Thus, we established the arsenite dose based on previous works showing that this concentration promotes proatherogenic dyslipidemias and hypertension in rats (31, 32). We also calculated the arsenic intake by measuring the water consumption of the animals (**Supplementary Figure 1A**). In our model, the rats treated with A and A+S consumed 4.19 ± 0.69 and 5.03 ± 0.99 mg of arsenic/kg of body weight/day (**Supplementary Figures 1B, C**), which is close to the non-observed adverse effect level calculated for orally ingested trivalent arsenic in rats (5 mg/kg/day) and is similar to the doses that induce insulin resistance and non-alcoholic fatty liver disease in rat models (33–36).

All animals were fed *ad libitum* with a standard chow diet for rats (Lab Diet 5001), as previously reported (37). We replaced drinking water with different treatments three times per week to prevent the growth of microorganisms and prevent arsenite oxidation. The treatments lasted for 8 weeks. All measurements and experiments were performed in the animals after fasting for 13 hours (8:00 PM to 9:00 AM). During this period, the treatments were replaced by plain water.

For determinations done during fasting (six animals for each condition), rats were anesthetized with an intraperitoneal injection of sodium pentobarbital (40 mg/kg) prior to the dissection of tissues. For determinations after insulin stimulation (20 animals for each condition, 10 stimulated with vehicle and 10 stimulated with insulin), fasted animals were given an intraperitoneal injection with 0.2 UI/kg of human insulin (Humulin^®^, Eli Lilly and Co., México) or an equivalent volume of sterile water (vehicle, less than 0.2 mL per rat). The animals were anesthetized after 30 min of stimulation. The quadriceps and the lateral head of the gastrocnemius muscles were excised, weighted, and quickly frozen in dry ice. Tissues were stored at -70°C until needed.

### Somatometric and biochemical parameters

We monitored the body weight of the animals every week and before sacrifice. The animal’s length was measured from the tip of the nose to the anus, and this data was used along with the final body weight to calculate the body mass index (BMI) (37). Blood fasting glucose concentration was measured by drawing blood from the tail vein before anesthetizing the animals and evaluated with a hand glucometer (Accu-Check, Hoffman La Roche, Basel, Switzerland). After sacrifice, the blood was drawn from the inferior cava vein into heparinized tubes, and plasma was isolated by centrifuging blood samples at 2000 rpm for 15s min at 4°C. Triglyceride levels were assessed using colorimetric glycerol phosphate oxidase and phenol 4-aminoantipyrene methods, using a Randox RX Imola, according to the manufacturer’s protocol. Insulin levels in plasma were assessed with an Ultrasensitive rat insulin ELISA system according to the manufacturer’s instructions (10-1137-10; Mercodia Uppsala, Sweden).

### Insulin tolerance test

We performed an ITT one week before the end of the treatment, as previously described (37). Briefly, 12 h-fasted rats received an intraperitoneal injection with 0.2 IU/kg of Humulin^®^ (Eli Lilly and Co., México). Blood samples were drawn from the tail vein immediately before the injection (time 0) and 15, 30, 60, 90, and 120 min after injection. Glucose concentrations were measured with a hand glucometer (Accu-Check, Hoffman La Roche, Basel, Switzerland). Animals that suffered from distress or that were not properly injected were eliminated from the analysis.

### Preparation of whole muscle lysates

Approximately 500 mg of muscle tissue were minced while still frozen and thawed in RIPA buffer (140 mM NaCl, 10 mM Tris-HCl pH 8.0, 1 mM EDTA, 1 mM EGTA, 1% Triton X-100, 0.1% sodium deoxycholate, and 0.1% SDS) freshly supplemented with cOmplete™, Mini Protease Inhibitor Cocktail (Roche) and 1 mM of sodium fluoride. We sonicated samples three times for 15 seconds each. Then, we centrifuged the samples at 10,000 X g for 18 min at 4°C. Supernatants were transferred to new tubes and the protein concentration was quantitated using the DC™ protein assay kit (Bio-Rad Laboratories, Hercules, CA US). The lysates were stored at -70°C until needed.

### GLUT4 translocation to sarcolemma and T-tubules

Sarcolemma fractions were obtained as described (38–40). Briefly, the lateral gastrocnemius and quadriceps muscles from insulin-stimulated rats were excised, and 0.5 mg of muscle was minced and quickly placed on 5 mL of ice-cold homogenization buffer (20 mM HEPES pH 7.4, 1 mM EDTA, 250 mM sucrose) freshly supplemented with protease inhibitors. Tissues were homogenized with a PRO250 tissue homogenizer (PRO Scientific, Monroe, CT USA) with three bursts of 10 seconds each. Homogenates were centrifuged in a benchtop centrifuge at 2000 X g for 10 minutes. The supernatant was transferred to new tubes and centrifuged in a JA-20.1 rotor at 9000 X g for 20 min. Then, the supernatant was centrifuged again in a 50Ti rotor at 180,000 X g for 90 min. All centrifugation steps were performed at 4°C. The pellet was resuspended in RIPA buffer and quantitated as described for whole muscle lysates.

### Quantitative immunoblot

Equal amounts of protein were mixed with 2X Laemli buffer and heated for 5 min at 85°C. Samples were loaded onto 12% SDS-PAGE and transferred at 15 V for 1 hour in a semi-dry chamber (Bio-Rad Laboratories, Hercules, CA US) to Immobilon-P, PVDF membranes (Millipore, MA, USA). Membranes were blocked for 1 hour at room temperature (RT) with tris-buffered saline + 0.1% Tween-20 (TBS-T) and 4% Blotto non-fat dry milk (Santa Cruz Biotechnology, Dallas, TX USA). Primary antibodies were incubated in TBS-T + 4% milk at 4°C overnight, according to the conditions described in **Supplementary Table 1**. The membranes were washed three times with TBS-T and incubated with the corresponding secondary antibody in TBS-T + 4% milk for 1 h at room temperature (horseradish peroxidase (HRP) goat anti-mouse dilution factor 1:4000, cat: sc-2005 Santa Cruz Biotechnology; and Peroxidase IgG Fraction Monoclonal Mouse Anti-Rabbit IgG, light chain specific, dilution factor 1:6000, cat: 211-032-171 Jackson Immunoresearch). To establish quantitative detection systems for each protein, we performed curves loading different amounts of total muscle lysate from a control rat. Then, the linearity of the detection system for each antibody was established (**Supplementary Figure 2**) (41). For each protein, the best running conditions were chosen based on these curves and are summarized in **Supplementary Table 1**. The blots were developed with ECL Prime Western Blotting Detection Reagent (GE Healthcare Life Sciences, Chicago, IL USA) in a C-Digit scanner (LI-COR Biosciences, NE USA). We used the optical density of the entire lane in the gels stained with Coomassie brilliant blue (CBB) as a loading control, as previously described (20). The immunoblots were analyzed using the Image Studio Lite ver. 5.2 Software (LI-COR Biosciences, NE USA). The results are presented as fold changes relative to the protein abundance observed in control animals.

### Cell culture and transfections

COS7 cells were obtained from the American Type Culture Collection (ATCC) and cultured as previously described (42). pcDNA3.1 plasmids containing CAPN10 isoforms a and c were gifted by Dr. Yasuko Ono (42). pTT3 plasmid containing SORT1-bio-His was a gift from Gavin Wright (Addgene plasmid # 52024). Cells were seeded in 3.5 cm dishes and transfected with 1.5 µg of the corresponding plasmid using the TransIT-X2 transfection system (Myrus Bio). Cells were collected 24 h after transfection and processed for Western blot as previously described (42).

### Statistical analysis and image assembly

All the experiments and determinations were done in at least four animals for each condition. Data analysis was done with GraphPad Prism 8.0 software. For all the experiments, we performed two-way ANOVA with Tukey’s *post hoc* test, with differences at p<0.05 considered statistically significant. Graphs present the mean ± standard error of the mean (SEM), and each circle denotes the individual animals used. The final figures were assembled using Adobe Photoshop 2022.

## Results

### Sucrose and arsenite induce metabolic syndrome signs

**Figure 1:**
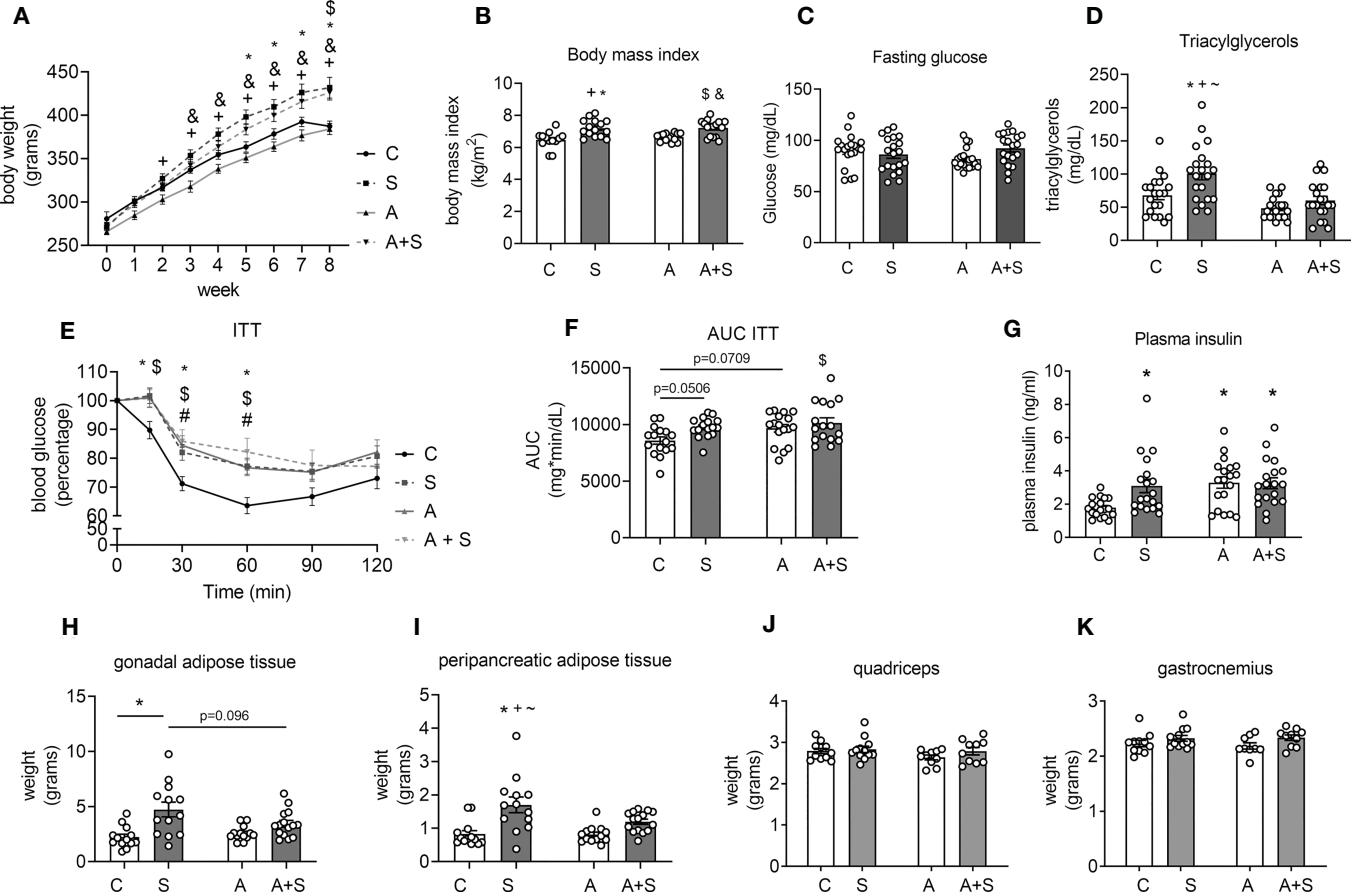
Characterization of the effects of sucrose and arsenic intake on parameters related to metabolic syndrome. **(A)** Weekly changes in body weight during the treatment of 8 weeks (n= 14 C, 15 S, 16 A, 17 A+S). **(B)** Body mass index at the end of treatment (n= 14 C, 15 S, 16 A, 17 A+S). **(C)** Blood fasting glucose (n= 20 animals in each condition). **(D)** Plasma triacylglyceride levels (n=20 animals in each condition). **(E)** Insulin tolerance test (ITT) was performed in the 7^th^ week of treatment (n= 16 C, 16 S, 17 A, 17 A+S). **(F)** Area under the curve (AUC) calculated from the ITT. **(G)** Plasma insulin levels in fasted animals (C= 19 in each condition). **(H–K)** Gonadal and peripancreatic adipose tissue weight (n= 13 C, 13 S, 13 A, 15 A+S). **(I, J)** Quadriceps and gastrocnemius weight (n= 11 C, 12 S, 10 A, 11 A+S). The graphs present the mean ± S.E.M., and individual animals are expressed as white circles. All data were analyzed by two-way ANOVA with Tukey’s *post hoc* test, and statistically significant differences were considered when p<0.05. ^*^S vs C, ^+^S vs A, ^#^A vs C, ^$^C vs A+S, ^&^A vs A+S, ^~^S vs A+S.

Rats treated for 8 weeks with sucrose (S) and arsenite + sucrose (A+S) gained more body weight compared with control (C) and arsenite (A)-treated rats (**Figure 1A**). Likewise, the body mass index (BMI) of animals in the 8th week of treatment with S and A+S was significantly higher than C and A-treated rats (**Figure 1B**). Thus, in our model, arsenic intake does not affect body weight gain induced by sucrose ingestion.

Neither S, A, nor A+S treatments altered fasting glucose blood levels compared with the control (**Figure 1C**). Sucrose consumption increased triacylglyceride plasma levels, but this effect was prevented in animals consuming A+S (**Figure 1D**). During the ITT, rats treated with S and A+S had statistically significantly higher blood glucose levels at 15 minutes. At the same time, S, A, and A+S displayed higher levels at 30 and 60 min of the ITT (p<0.05), displaying an increased area under the curve (AUC; C vs S p=0.0506, C vs A p=0.0709 and C vs A+S p<0.05), indicating that all treatments induced insulin resistance (**Figures 1E, F**). Consistent with the presence of insulin resistance observed after the ITT, all treatments increased plasma insulin levels compared with the control (**Figure 1G**). The evidence shows that sucrose induces at least three of the MS signs (obesity, hypertriglyceridemia, and insulin resistance), while arsenic only favored the development of insulin resistance and prevented the hypertriglyceridemia induced by sucrose without altering body weight gain.

Interestingly, the weight and the percentage of body weight of the peripancreatic and gonadal adipose tissues increased in S-treated animals but not in A+S, compared with C and A (**Figures 1G, H**, **Supplementary Figure 3**). The weight and the percentage of body weight of the quadriceps and gastrocnemius muscles was not different between the groups (**Figures 1I, J**, **Supplementary Figure 3**).

### Arsenic and sucrose altered GLUT4 trafficking to the sarcolemma

Since glycolytic muscles, such as quadriceps and gastrocnemius are more susceptible to metabolic dysfunction induced by high-sucrose diets (43), and these muscles are among the largest muscle groups in rats, we studied how insulin signaling is affected by the treatments in these muscles. We obtained sarcolemma fractions from the quadriceps and gastrocnemius muscles 30 min after intraperitoneal injection with 0.2 IU/kg insulin to evaluate the abundances of GLUT4 and GLUT1 (the glucose transporter constitutively present at the sarcolemma).

**Figure 2:**
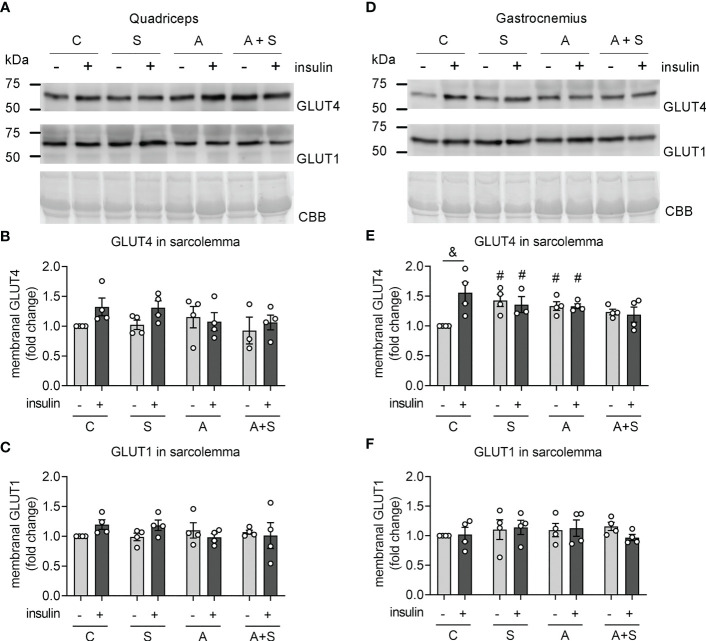
Effects of sucrose and arsenic intake on GLUT4 abundance in the sarcolemma. We determined the protein abundances of GLUT4 and GLUT1 in sarcolemma fractions 30 min after stimulation with an intraperitoneal injection of 0.2 IU/kg of insulin. **(A–C)** Results in quadriceps. **(D–F)** Results in gastrocnemius. **(A, D)** Representative Western blot. **(B, E)** Quantitation of GLUT4 levels in the sarcolemma. **(C, F)** GLUT1 was used as a negative control of a protein constitutively expressed at the sarcolemma. The graphs present the mean ± S.E.M. of four animals for each condition. Individual animals are expressed as white circles. All data were analyzed by two-way ANOVA with Tukey’s *post hoc* test, and statistically significant differences were considered when p<0.05. ^&^Effect of insulin. ^#^Effect of treatment, compared with Control without insulin.

Neither insulin stimulation nor treatments altered GLUT4 and GLUT1 abundances in sarcolemma fractions from the quadriceps (**Figures 2A–C**), indicating that this muscle group has low insulin sensitivity, at least under our conditions. In contrast, insulin stimulation increased GLUT4 abundance in the sarcolemma from the gastrocnemius in control animals (**Figures 2D, E**). Moreover, S and A induced a statistically significant increase in basal GLUT4 levels, while S, A, and A+S blunted insulin-stimulated GLUT4 translocation (**Figures 2D, E**), correlating with the results from the ITT.

As expected, GLUT1 levels in sarcolemma fractions did not change due to insulin and treatments in gastrocnemius (**Figure 2F**). These results indicate that S, A, and A+S specifically affect GLUT4 trafficking induced by insulin while impairing basal GLUT4 intracellular retention in gastrocnemius.

### Arsenite and sucrose impair the expression of GSV markers

**Figure 3:**
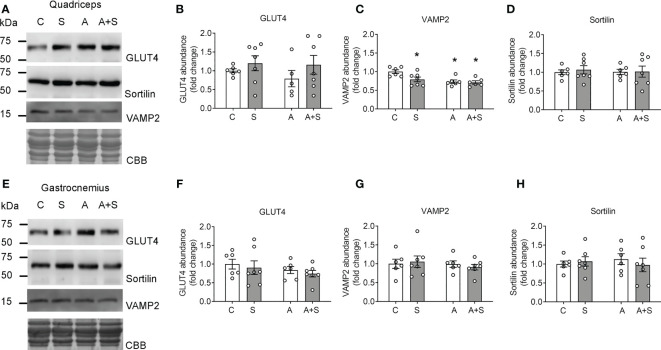
Sucrose and arsenic impair GSV markers in the quadriceps but not in the gastrocnemius. Total muscle lysates were prepared from animals after overnight fasting. **(A–D)** Results in quadriceps. **(E–H)** Results in gastrocnemius. **(A, E)** Representative Western blot. **(B, F)** Quantitation of total GLUT4 levels. **(C, G)** VAMP2 quantitation. **(D, H)** Sortilin quantitation. The graphs present the mean ± S.E.M. of six animals for each condition. Individual animals are expressed as white circles. All data were analyzed by two-way ANOVA with Tukey’s *post hoc* test, and statistically significant differences were considered when p<0.05. *vs Control animals.

Defects in insulin-stimulated GLUT4 translocation during insulin resistance can be due to impaired sorting of GLUT4 into the GSVs and the downregulation of regulatory proteins of GSV trafficking, such as VAMP2 and sortilin (44–47). Thus, we tested whether the effects observed in GLUT4 abundance in the sarcolemma could be related to changes in total GLUT4, VAMP2, and sortilin levels. The total abundance of GLUT4 was unchanged under all conditions in both muscles, demonstrating that the alterations observed in GLUT4 abundance in the sarcolemma were due to impaired signaling and trafficking, rather than changes in GLUT4 expression (**Figures 3B, F**). Conversely, protein levels of VAMP2 (a member of the v-SNARE family of proteins, which is important for GSV docking and fusion with the plasma membrane (18)) decreased in the quadriceps muscles from animals treated with S, A, and A+S, while there were no effects in the gastrocnemius (**Figures 3C, G**). Sortilin is an important protein for GLUT4 recruitment into the GSVs (46). Nevertheless, we did not find differences in the levels of this protein in both muscles (**Figures 3D, H**).

Intriguingly, the band detected by the sortilin antibody in both muscles was around 60 kDa, which is lower than the expected molecular weight of 92 kDa. We validated that this signal was, in fact, sortilin by exogenously expressing human sortilin in COS7 cells and comparing the signal with those observed in rat muscles (**Supplementary Figure 4**). As expected, COS7 cells transfected with the plasmid containing human sortilin expressed two bands of approximately 100 and 90 kDa, corresponding to pro-sortilin and its mature form. In contrast, both muscles expressed only the 60 kDa form of insulin at levels compared with the exogenously expressed sortilin in COS7 cells, thereby demonstrating that this signal is, in fact, an isoform of sortilin. Nevertheless, the entries for the rat, mouse, and human sortilin gene (accession numbers: 83576, 20661, and 6272, respectively) show only one isoform (100 kDa) in rats, two isoforms (91 and 87 kDa) in mice, and two isoforms (88 and 77 kDa) in humans. Thus, the structure of this muscle isoform in rats remains to be determined.

### Arsenite, but not sucrose, inhibits Akt phosphorylation

Since no defects in GSV markers in the gastrocnemius could explain the alterations observed in GLUT4 trafficking, we next tested whether the treatments affected the abundance of Akt and insulin-stimulated Akt phosphorylation at serine residue 473, which is a marker of fully active Akt (48).

**Figure 4:**
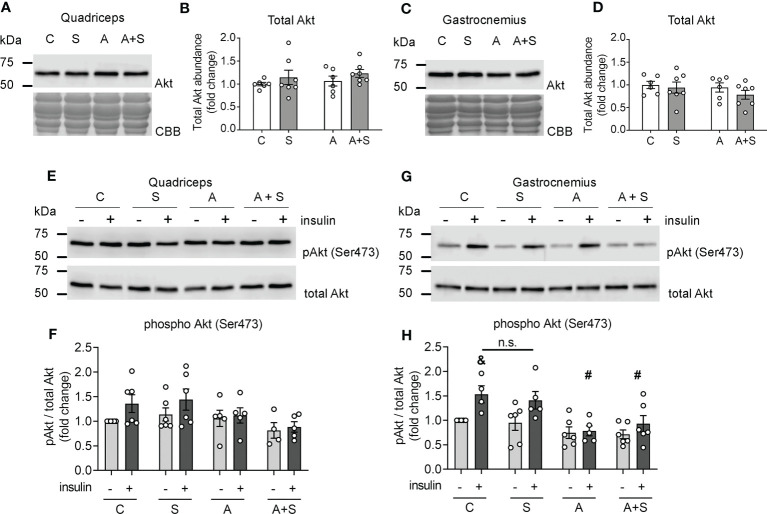
Arsenite impairs Akt phosphorylation induced by insulin. **(A–D)** Total lysates from fasted rats were used for total Akt determination in quadriceps **(A, B)** and gastrocnemius **(C, D)** muscles. **(E–H)** Lysates from rats stimulated with vehicle or insulin were used to evaluate phosphor Akt (Ser473) levels in quadriceps **(E, F)** and gastrocnemius **(G, H)**. The graphs present the mean ± S.E.M. of six animals in each condition. Individual animals are expressed as white circles. All data were analyzed by two-way ANOVA with Tukey’s *post hoc* test, and statistically significant differences were considered when p<0.05. ^&^Insulin vs the same treatment with vehicle. ^#^vs Control with insulin. n.s. stands for non-significant.

Neither treatment altered the abundance of total Akt protein in the quadriceps nor the gastrocnemius muscles (**Figures 4A–D**). Comparable to the changes in GLUT4 abundance in the sarcolemma, insulin injection induced Akt phosphorylation in the gastrocnemius but not the quadriceps from control animals (**Figures 4E–H**). Interestingly, insulin-induced Akt phosphorylation did not change in the gastrocnemius from S-treated animals (**Figures 4G, H**), while arsenite blunted insulin-stimulated pAkt levels independently of sucrose intake (**Figures 4G, H**). No changes in pAkt levels were observed in the quadriceps after any treatment (**Figures 4E, F**).

### Sucrose and arsenite intake have different effects on capn10 and TUG proteolysis

Insulin promotes TUG proteolysis and dissociation of TUG-GLUT4 complexes, releasing the GSVs (23). Moreover, TUG’s N- and C-terminal fragments promote GSV trafficking and expression of thermogenic genes, respectively (21, 23). Thus, we evaluated the possible alterations in the two proteases that perform TUG proteolysis [capn10 and usp25m (20, 21)], as well as changes in the abundance of intact TUG and its proteolytic C-terminal fragments..

**Figure 5:**
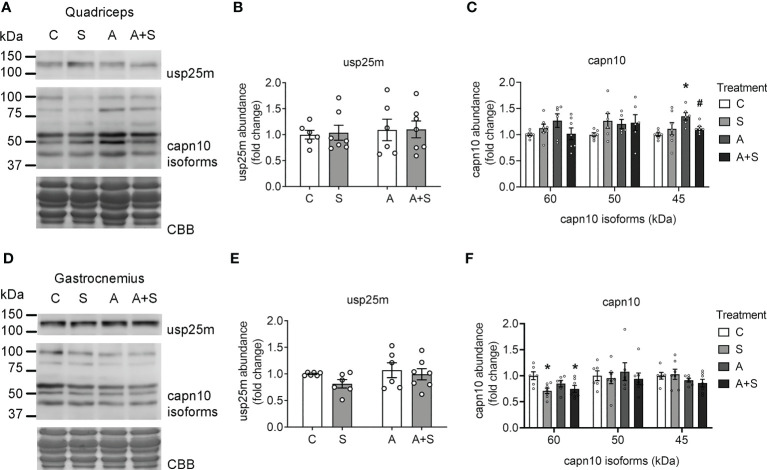
Sucrose and arsenic intake affect CAPN10 abundance. Total lysates from fasted rats were used to quantitate the levels of Usp25m and capn10 proteases in quadriceps **(A–C)** and gastrocnemius **(D–F)**. The graphs present the mean ± S.E.M. of six animals in each condition. Individual animals are expressed as white circles. All data were analyzed by two-way ANOVA with Tukey’s *post hoc* test, and statistically significant differences were considered when p<0.05. * vs control, ^#^ vs A.

We did not find changes in the abundance of the protease usp25m under any conditions in either the quadriceps or the gastrocnemius (**Figures 5A, B, D, E**). In both muscles, capn10 was detected as three main putative isoforms (60, 50, and 45 kDa; the 60 kDa isoforms being the most abundant in both muscles) and unidentified high molecular weight bands, as described previously (42, 49). In the quadriceps, the treatments altered neither the main isoform of 60 kDa nor the 50 kDa isoform. However, A-treated animals displayed higher levels of capn10 45 kDa isoform compared with the control, and this increase was prevented in A+S (**Figure 5C**). In contrast, in the gastrocnemius muscle from S and A+S-treated animals, the abundance of capn10 60 kDa isoform was significantly reduced compared with the control animals, without changes in the 50 and 45 kDa isoforms (**Figure 5F**). Of note, by comparing the rat capn10 isoforms present in these muscles with exogenously expressed human CAPN10a and CAPN10c isoforms in COS7 cells, it can be reasonably concluded that the predominant capn10 isoform present in muscle (60 kDa isoform) corresponds to isoform c (capn10c). In comparison, the capn10 bands at 50 and 45 kDa could be proteolytic fragments or unknown splicing isoforms (**Supplementary Figure 5**).

**Figure 6:**
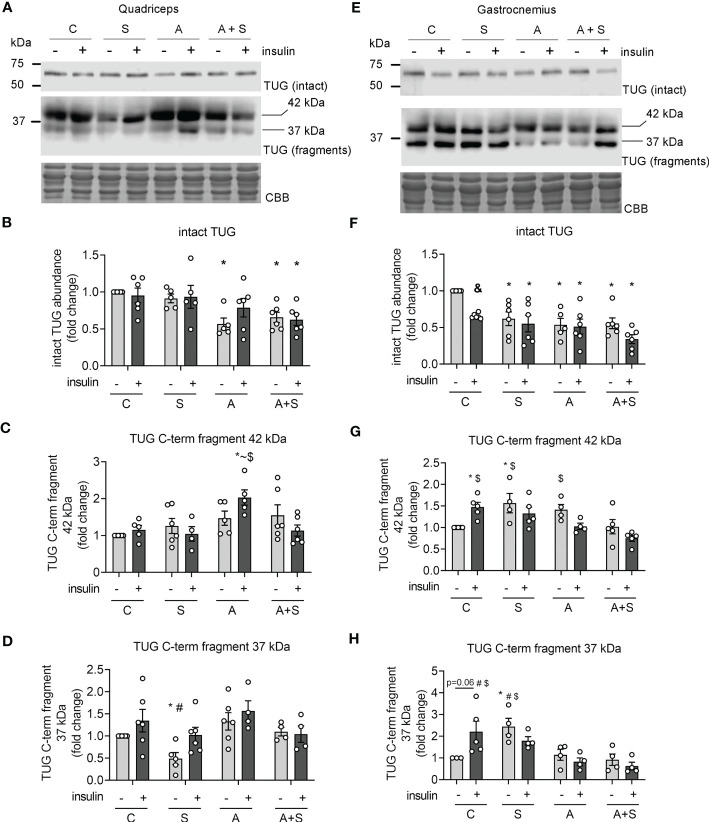
Effects of arsenic and sucrose intake on TUG proteolysis. Lysates from rats stimulated with vehicle or insulin were used to evaluate the abundance of intact TUG and its C-terminal fragments in quadriceps **(A–D)** and gastrocnemius **(E–H)**. The graphs present the mean ± S.E.M. of six animals in each condition. Individual animals are expressed as white circles. All data were analyzed by two-way ANOVA with Tukey’s post hoc test, and statistically significant differences were considered when p<0.05. * vs control without insulin, ~ vs S insulin, ^#^ vs A, ^$^ vs A+S insulin, ^&^ Insulin vs the same treatment with vehicle.

Next, we measured the changes in the abundance of intact TUG after 30 min of insulin stimulation, as a marker of TUG proteolysis (21, 23). Concordantly with the lack of response to insulin stimulation in the quadriceps at the levels of GLUT4 translocation and Akt phosphorylation, the levels of intact TUG were not altered after insulin stimulation in the control animals (**Figures 6A, B**). Nevertheless, the arsenic treatment decreased basal and insulin-stimulated TUG abundance, regardless of sucrose intake (**Figures 6A, B**). In contrast, TUG abundance in the gastrocnemius muscle from control animals was significantly reduced after insulin stimulation (**Figures 6E, F**), which is consistent with the loss of intact TUG due to insulin-stimulated proteolysis. In this muscle, S, A, and A+S reduced the basal abundance of TUG and completely abolished insulin-stimulated TUG proteolysis (**Figures 6E, F**). To further characterize the deregulation of TUG proteolysis, we evaluated the abundance of the proteolytic fragments of this protein. In our model, we found two main fragments containing the C-terminal domain of TUG of 42 and 37 kDa (**Figures 6A, B**). In the quadriceps, the 42 kDa fragment was induced in animals exposed to arsenite and stimulated with insulin (**Figures 6A, C**), while the fragment of 37 kDa was reduced in animals exposed to sucrose during fasting, but this was reversed after insulin stimulation (**Figures 6A, D**). In the gastrocnemius, insulin increased the abundance of both fragments in control animals (**Figures 6E, G, H**). Interestingly, the levels of the 42 kDa fragment increased in S and A animals but not in A+S animals, independently of the stimulation with insulin (**Figures 6E, G**). This effect was not observed in the abundance of the fragment of 37 kDa in arsenite-treated animals, but this fragment was increased in S-exposed animals during fasting but not after insulin stimulation (**Figures 6E, H**).

## Discussion

Skeletal muscle plays a pivotal role in controlling whole-body glucose homeostasis, and muscle insulin resistance is a major factor in the pathophysiology of MS and T2D (18). In the present study, we compared the effects of sucrose and arsenic intake for two months in developing the signs of MS, along with the alterations in muscle insulin signaling pathways that control GLUT4 translocation to the sarcolemma.

Our results showed that both sucrose and arsenite induced whole-body insulin resistance, even when arsenite did not modify body weight and prevented hypertriglyceridemia induced by sucrose. Interestingly, sucrose and arsenite had no additive effects on insulin resistance. Based on the calculations of the arsenic intake in our model (4.19 ± 0.69 and 5.03 ± 0.99 mg/kg/day), we can compare our data with previous reports of insulin resistance and hyperinsulinemia induced by intraoral administration of arsenite in male rats (2.5-5 mg/kg/day) (34, 36). Thus, it is appropriate to state that arsenic exposure in the range of 2.5-5 mg/kg/day induces insulin resistance without the development of obesity, at least in rat models.

On the other hand, arsenite did not affect the increase in body weight induced by sucrose intake, but it prevented adipose tissue hypertrophy of the two adipose tissue depots that we analyzed. These discrepancies could be related to arsenic-induced lipodystrophy, resulting in the lower ability of adipose tissue to handle an excess of calories and favoring ectopic lipid deposition in other tissues (2, 34, 50). Thus, our results indicate that arsenite affects the development of MS traits induced by sugary drinks in different ways.

Skeletal muscle accounts for up to 80% of the total glucose disposal during insulin stimulation, and defective GLUT4 trafficking in skeletal muscle is a major factor in developing MS and T2D (18). Since all treatments reduced insulin-induced glucose disposal during the ITT, we tested whether arsenite and sucrose affect some of the pathways involved in GLUT4 trafficking to the sarcolemma.

In the quadriceps, insulin stimulation (0.2 IU/kg) did not increase GLUT4 abundance in the sarcolemma, nor promote Akt phosphorylation and TUG proteolysis. Moreover, neither arsenite nor sucrose altered the levels of GLUT4 in the sarcolemma and phospho-Akt in this muscle group. Nevertheless, all treatments diminished VAMP2 levels, and arsenite and sucrose + arsenite diminished the basal levels of TUG, but this latter effect was probably due to enhanced TUG proteolysis and lower gene expression, respectively. Although low TUG levels result in impaired intracellular retention of GLUT4 and high levels of GLUT4 in the sarcolemma during fasting (23), we did not find changes in GLUT4 levels in the sarcolemma in the quadriceps from arsenic-treated rats.

Although the lack of insulin response in quadriceps might seem paradoxical, most studies on GLUT4 translocation in muscle use high insulin doses (about 2 IU/kg), which are 10 times higher than the insulin doses used for the treatment of T2D patients and the dose used for this study (0.2 IU/kg) (2, 21, 51). Moreover, different muscle groups have distinct insulin sensitivity, especially at submaximal concentrations (52). Therefore, we propose that the quadriceps muscle has low insulin sensitivity in rats, and alterations related to GSV trafficking include reduced VAMP2 after sucrose and arsenite treatment and decreased TUG abundance after arsenic treatment.

In contrast, insulin induced a ~1.5-fold increase in GLUT4 levels in the sarcolemma from gastrocnemius, concordant with the expected increase of membranal GLUT4 described in skeletal muscle (18). Likewise, insulin stimulated Akt phosphorylation and TUG proteolysis in this muscle. In parallel with systemic insulin resistance, all treatments blunted insulin-induced GLUT4 recruitment to the sarcolemma in the gastrocnemius. Interestingly, only arsenite treatment impaired Akt phosphorylation, independently of sucrose intake, while all treatments dulled TUG proteolysis induced by insulin in the gastrocnemius. It is of note that the levels of the proteolytic fragments of TUG were differentially deregulated by treatments: sucrose increased the abundance of both fragments, arsenite only increased the fragment of 42 kDa, and arsenite + sucrose did not change the abundance of any fragment; thus, we hypothesize that sucrose and arsenite accelerate basal proteolysis of TUG, while TUG gene expression is impaired in animals consuming both factors. Whether this increase in basal TUG proteolysis is carried out by capn10 and usp25m or by other proteases that cleave this protein [such as calpain-1 and the ubiquitin-proteasome system (20, 23)] remains to be explored.

The results in the gastrocnemius muscle suggest that sucrose and arsenic intake impair GLUT4 trafficking in gastrocnemius through different pathways: while sucrose deregulates the proteolytic pathway, arsenic affects both proteolytic and PI3K-Akt pathways. However, Akt levels must be reduced by more than 90% to effectively impair GLUT4 translocation in skeletal muscle (16), and arsenic reduced Akt phosphorylation by ~60%. Also, reduced p-Akt levels due to hypercaloric diets do not necessarily correlate with reduced phosphorylation of its target proteins (TBC1D1 and TBC1D4) and GLUT4 translocation (17). Thus, we can hypothesize that impaired TUG proteolysis exerts a higher contribution to blunted GLUT4 translocation after arsenic and sucrose exposure.

Consistent with previous reports (10, 13, 17, 21), our data suggest that the proteolytic pathway is more sensitive to environmental cues than the PI3K-Akt pathway, and arsenic has a high impact on Akt phosphorylation, probably by inhibiting PDK-1 (14). Notably, we found higher levels of GLUT4 in the sarcolemma during fasting in the gastrocnemius from sucrose and arsenite-treated animals. As previously stated, low levels of TUG in skeletal muscle result in the defective retention of GLUT4 in intracellular compartments (23). Therefore, the high fasting levels of GLUT4 at the sarcolemma could be due to the enhanced basal TUG proteolysis in animals consuming sucrose and arsenite separately and the low expression of TUG in animals consuming both factors. We hypothesize that the impaired intracellular retention of GLUT4 in this muscle could contribute to maintaining normal blood glucose levels, even in insulin-resistant states.

Although the deregulation of the proteolytic pathway is an important factor contributing to insulin resistance in muscle, the precise upstream mechanisms that regulate this pathway under physiological conditions remain largely unknown. Interestingly, high-fat dietary intake reduces the protein levels of the protease usp25m in the quadriceps of mice (23). Nevertheless, we did not find changes in the levels of this protease after sucrose and arsenic exposure in rats in any of the muscles analyzed. We found that capn10 isoforms were differentially deregulated in the quadriceps and gastrocnemius muscles. Whether these discrepancies could be due to species-specific mechanisms or the differences in the source of calory surplus (fat vs sucrose) remains to be determined in future studies. Nevertheless, neither change in capn10 isoforms, nor usp25m levels fully explain why all treatments inhibited TUG proteolysis. Further research is needed to understand the precise mechanisms that activate these proteases in skeletal muscle and how environmental and dietary factors affect their activity.

## Conclusion

Our results indicate that arsenic and sucrose induce systemic insulin resistance related to defects in the translocation of GLUT4 to the sarcolemma in skeletal muscle. Importantly, sucrose and arsenic do not have additive effects in developing insulin resistance. The defects in the pathways involved in this process depend on the muscle analyzed and the environmental factors. In the quadriceps, the main alterations were related to markers of GSV intracellular retention and docking. At the same time, in the gastrocnemius, sucrose altered the proteolytic pathway and arsenic impaired Akt phosphorylation and TUG proteolysis.

## Data availability statement

The raw data supporting the conclusions of this article will be made available by the authors, without undue reservation.

## Ethics statement

The animal study was reviewed and approved by Animal Care Committee of the Instituto de Fisiología Celular, Universidad Nacional Autónoma de México (UNAM; CICUAL MHU189-22).

## Author contributions

PP: conceptualization, experimental design, investigation, data curation, draft writing and editing, visualization. MV: experimental design, investigation, data curation, manuscript review and editing. AS: experimental design, manuscript review and editing, funding acquisition. POW: conceptualization, experimental design, manuscript review and editing, funding acquisition. MH: conceptualization, experimental design, manuscript review and editing, funding acquisition, project management. All authors contributed to the article and approved the submitted version.
