# Supplementary material for: The effects of sucrose and arsenic on muscular insulin signaling pathways differ between the gastrocnemius and quadriceps muscles
Source: Front Endocrinol (Lausanne). 2023 May 9;14:1165415. doi: 10.3389/fendo.2023.1165415 (PMC10205014; doi:10.3389/fendo.2023.1165415)
Supplement: Supplementary file 1 [file DataSheet_1.docx]

Supplementary Table 1. Antibodies used for Western blot analysis.

| Protein | Primary antibody (catalog and brand) **Dilution factor** | Amount of lysate (µg of protein) |
| --- | --- | --- |
| GLUT4 | Mouse anti-GLUT4 (IF8) (cat: sc-53566, Santa Cruz Biotechnology)  **1:1000** | 10 µg total lysates  80 µg sarcolemma fractions |
| GLUT1 | Mouse anti-GLUT1 monoclonal (cat: LS-C129284, LifeSpan BioSciences)  **1:1000** | 80 µg sarcolemma fractions |
| VAMP2 | Rabbit anti-VAMP2 (EPR12790) (cat: ab198949, Abcam) **1:500** | 40 µg total lysate |
| Sortilin | Mouse anti-Sort1 (cat: MA5-31437, Thermo Fisher Scientific) **1:1000** | 20 µg total lysate |
| Akt | Rabbit anti-Akt (pan) (11E7) (cat: 4685, Cell Signaling Technology) **1:2000** | 40 µg total lysate |
| pAkt (Ser473) | Rabbit anti-phospho Akt (Ser473) (cat: 9271, Cell Signaling Technology) **1:1000** | 40 µg total lysate |
| Usp25m | Rabbit anti-Usp25 (EPR15019)(cat: ab187156, Abcam) **1:2000** | 40 µg total lysate |
| Calpain-10 | Rabbit anti-calpain-10 domain III (cat: ab28226, Abcam) **1:1000** | 40 µg total lysate |
| TUG | Rabbit anti-TUG (cat: 2049, Cell Signaling Technology) **1:500** | 40 µg total lysate |
| TUG C-term  fragment | Rabbit anti-TUG (cat: 2049, Cell Signaling Technology) **1:500 (secondary Ab 1:3000)** | 40 µg total lysate |

**Supplementary Figure 1. Water and arsenic intake.** We measured the water intake the same day when the animals were weighted. Then, total arsenic ingestion was calculated as mg of arsenic/kg of body weight/day. Data were analyzed with t-test. *p<0.05.


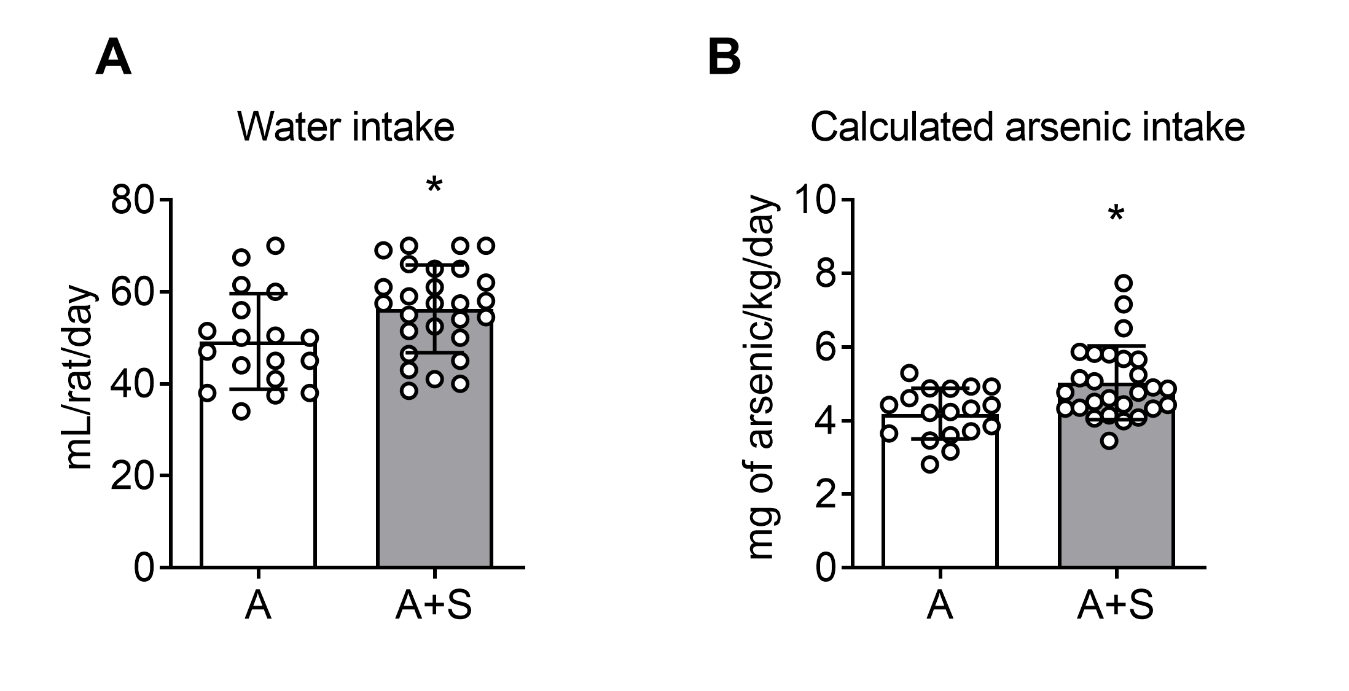


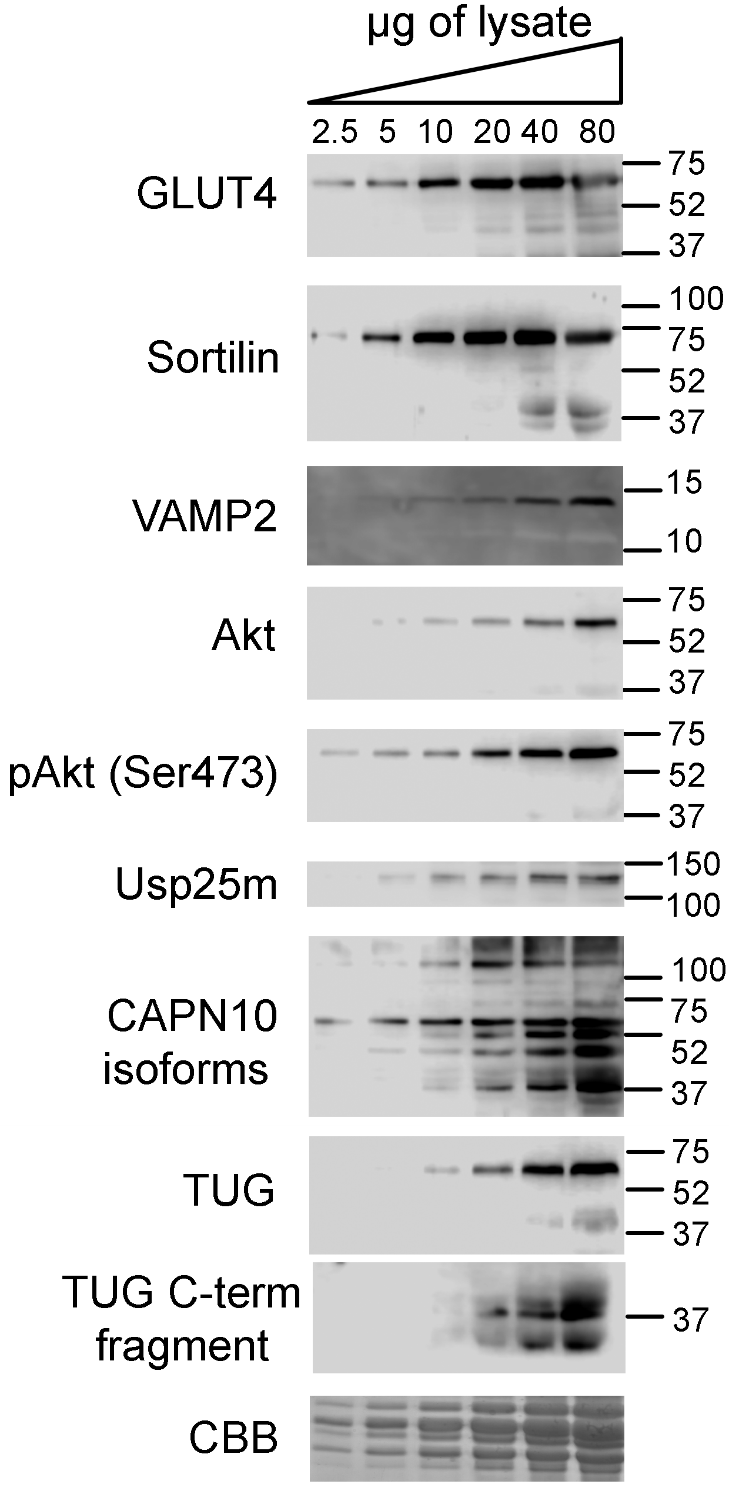
**Supplementary Figure 2.** Validation of the quantitative immunoblot for each of the antibodies tested during the present study. We performed curves ranging from 2.5 to 80 µg of protein from Quadriceps lysates from a control animal. The linearity of the detection system was further validated by linear regression analyses.

**Supplementary Figure 3. Percentage of tissue weight relative to total body weight.** The weight of the tissues in Figure 1 was normalized as percentage to total body weight of each animal. Data were analyzed by two-way ANOVA and Tukey’s post-hoc test and statistically significant differences were considered when p<0.05. * S vs C, + S vs A, ~ S vs A+S.





**

Supplementary Figure 4**. Validation of the sortilin detection by Western blot was performed by comparing the signal obtained from rat skeletal muscles with the exogenous overexpression of human-Sort1 in COS7 cells. For sortilin detection in COS7 cells, we loaded 20 µg of total lysate. For skeletal muscle samples, we loaded 10 µg. Black arrowhead denotes pro-sortilin gene product, white arrowhead: mature sortilin. Gray arrow: Sortilin isoform detected in skeletal muscle.

**Supplementary Figure 5.** Validation and comparison of the CAPN10 expression in quadriceps and gastrocnemius muscles. A) Western blot of COS7 cells transfected with an empty vector or plasmids containing the sequence of splicing-resistant human CAPN10a (srCAPN10a) and human CAPN10c isoforms. Three independent samples of quadriceps and gastrocnemius muscles from control animals are included. * non-specific signal. B) comparison between the expression levels of the three putative CAPN10 isoforms in quadriceps and gastrocnemius muscles. The signal of the bands observed in muscles were adjusted to the signal observed in COS7 cells expressing CAPN10c. No significant differences were observed.
